# The applicability of the Greulich & Pyle Atlas for bone age assessment in primary school-going children of Karachi, Pakistan

**Published:** 2014

**Authors:** Arsalan Manzoor Mughal, Nuzhat Hassan, Anwar Ahmed

**Affiliations:** 1 Dr. Arsalan Manzoor Mughal, MBBS, M. Phil Candidate, Senior Lecturer, Department of Anatomy, Dr. Ziauddin Hospital, Ziauddin University, North Nazimabad, Karachi.; 2 Dr. Nuzhat Hassan, MBBS, M.Phil (Anatomy), Professor& Head of Anatomy Department, Dr. Ziauddin Hospital, Ziauddin University, North Nazimabad, Karachi.; 3 Dr. Anwar Ahmed, MBBS, FMRD, MD, FCPS, Associate Professor of Diagnostic Imaging, Dr. Ziauddin Hospital, Ziauddin University, North Nazimabad, Karachi.

**Keywords:** Bone Age Measurement, Diagnostic X-Ray Radiology

## Abstract

***Objective:*** To assess the degree of applicability of bone age calculated by Greulich & Pyle Atlas in estimation of chronological age for therapeutic and medico legal purposes.

***Methods: ***Two Hundred and Twenty children (139 males, 81 females) between ages of 56 and 113 months (4.5 to 9.5 years) were randomly selected from 4 primary schools of Shireen Jinnah & Clifton, Karachi. Digital images of hand and wrist radiographs were obtained by a computed radiography at Ziauddin Hospital Clifton. Bone ages were computed using Greulich & Pyle Atlas by radiologists at Ziauddin Hospital, North Nazimabad, Karachi.

***Results:*** On average, the Greulich & Pyle Atlas underestimates chronological age by 6.65 ± 13.47 months in females and 15.78 ± 12.83 months in males (p-values < 0.001). High correlation was found between chronological age and bone age in both genders (Females r=0.778; p-value< 0.001, Males r=0.816; p-value < 0.001).

***Conclusion: ***Bone age calculated by Greulich & Pyle Atlas should not be used for estimating chronological age in children of ages 56-113 months in situations where high accuracy is required (e.g. medicolegal cases). However, serial measurements of bone age by this atlas can be used in management of growth related endocrine disorders in these children.

## INTRODUCTION

The status of skeletal growth of children is often assessed by calculation of bone age. This age is different from the chronological age of a child, which is calculated from the date of birth. It is often used by pediatricians for the diagnosis of growth disorders and serial measurements are required during their treatment.^1^ Bone age is also requested in medico legal cases such as criminal trials^2^, immigration^3^ and sports^4^ to estimate the chronological age when the exact birth record of a child is not available. For these purposes, normal standards of bone age should accurately represent chronological age. An over or underestimation of bone age can result in the inappropriate diagnosis and treatment of growth disorders, unjust punishment, misplacement in a new school or undue advantage in competitive sports.

Various methods have been developed to compute bone age. One of the oldest, simplest^5^ and most frequently used method of bone age calculation in Pakistan is the Greulich & Pyle Atlas^6^. This atlas was developed by Dr. Wiliam Walter Greulich and Dr. Sarah Idell Pyle in 1959^6^ using data collected from the “Brush foundation study of human growth and development” headed by Professor Wingate Todd^7^ on Caucasian children of the upper socioeconomic class living in United states of America. The atlas comprises of reference radiographic images of left wrist and hand from birth till 19 years of age for males and 18 years of age for females. It is based on the fact that ossification centers in the hand and wrist bones appear in a sequential and fixed order. Bone age is calculated by comparing the degree of ossification in various hand and wrist bones with the nearest matching plate on the Greulich & Pyle Atlas separately for male and female children.

Due to the fixed ethnic and socioeconomic class of children selected for generation of this atlas, its applicability varies in different parts of the world. It is considered to compute a bone age which is comparable to chronological age in children from developed countries of West^8^ and Middle east^9^ but great difference exists between the two in children from developing countries such as Iran^10^ and India.^11^A couple of studies have been conducted to assess the reliability of Greulich & Pyle Atlas in Pakistani children. One of them has a retrospective study design which could result in recall bias,^12^ the other has limited the cohort to older children of ages 8-18 years.^13^ Thus bringing about the need to assess the usability of this method in younger Pakistani children with a relatively accurate date of birth acquired from a valid source of data.

## METHODS

School children between the ages 54 to 113 months (4.5 to 9.5 years) were randomly selected for the study from 4 different schools in the Clifton and Shireen Jinnah Colony Regions of Karachi. After approval from the school administration, camps were setup at school where parents and children were recruited. Consent forms were signed by parents, subsequently assent forms were signed by children and they were given an appointment at Ziauddin Hospital Clifton. At the hospital their height and weight were noted. Children with a major childhood illness lasting more than 3 months or height above 95^th^ percentile for age or height below 5^th^percentile for age were excluded from the study. Information regarding the date of birth of the included subjects was obtained from school records. A total of 244 subjects underwent radiography of the left wrist and hand (Posterior Anterior view) using a SHIMADZU radiographic system. Exposure was obtained on an AGFA cassette and digitalized by AGFA 30-X CR Reader.220 digital images of radiographs were selected and 24 were rejected due to inadequate exposure. Bone age was computed from the selected radiograph images using Greulich & Pyle Atlas by radiologists at Ziauddin Hospital, North Nazimabad campus. This study was approved by ethical committee of Ziauddin University and funds for the project were also provided by the aforesaid university.

Data was entered and analyzed using statistical software SPSS version 20. Paired student’s T-Test was used to compare paired differences between chronological ages and bone ages calculate by Greulich & Pyle Atlas. Pearson’s Correlation analysis was used to determine a relationship between these two ages. Bland Altman plots were generated to visualize agreement between the two ages. Results with p-value less than 0.05 were considered statistically significant.

## RESULTS

The mean difference between chronological age and bone age was less in females as compared to males (6.65 & 15.75 months respectively; p-values < 0.001). However, a strong significant positive correlation (r= 0.788 & 0.816 respectively; p-values, 0.001) was noted between chronological age and bone age in both the genders (Table-I).

**Fig.1:**
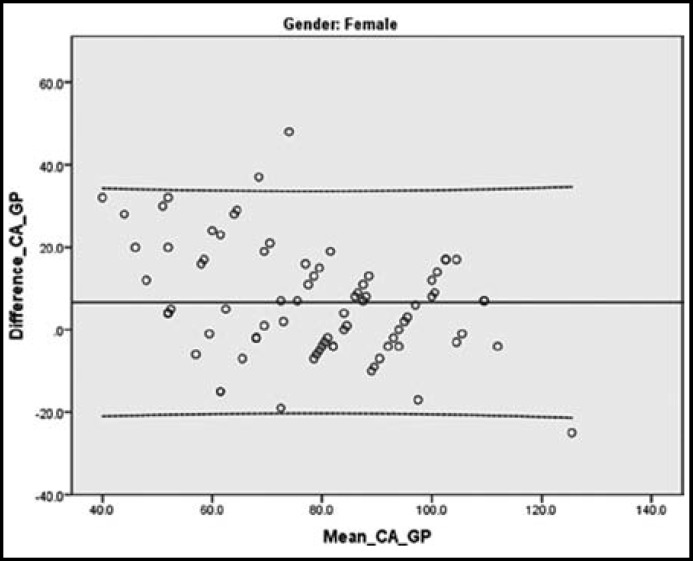
Bland Altman Plot (females). Solid lines represent mean of differences. Dotted lines represent 95% limits of agreement

Bland Altman Plot of females shows a mean difference of +6.65 months between chronological age and bone age. 95% of the points lie between -19.75 months and +33.05 months. 3.7% points lie beyond ± 2 SD lines.(Fig.1).

MD= Mean of differences, SD= Standard deviation,

Difference_CA_GP= chronological age(months)-Bone age by Greulich & Pyle Atlas(months)

Mean_CA_GP= chronological age(months)+Bone age by Greulich & Pyle Atlas(months)/2

MD= +6.65 months, MD + 2 SD= +33.05 months, MD - 2 SD= -19.75 months.

**Fig.2:**
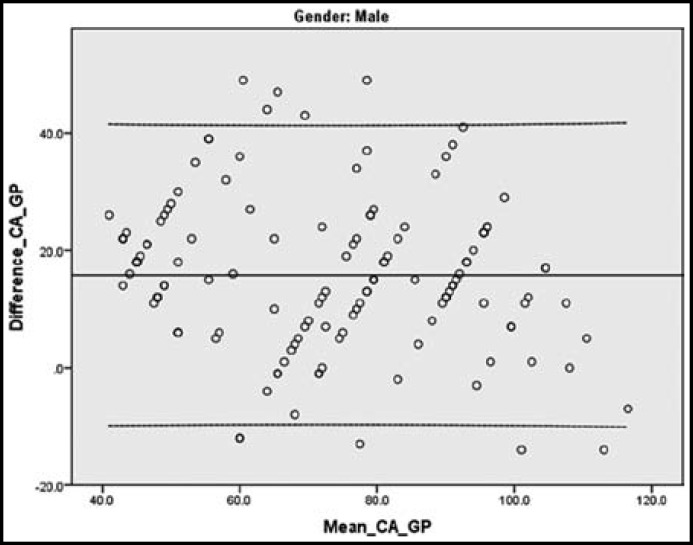
Bland Altman Plot (males). Solid lines represent mean of differences. Dotted lines represent 95% limits of agreement

Bland Altman Plot of males shows a mean difference of +15.78 months between chronological age and bone age. 95% of the points lie between - 9.37 months and +40.93 months. 7.2% points lie beyond ± 2 SD lines.(Fig.2).

,

MD= Mean of differences, SD= Standard deviation,

Difference_CA_GP= chronological age(months)-Bone age by Greulich & Pyle Atlas(months)

Mean_CA_GP= chronological age(months)+Bone age by Greulich & Pyle Atlas(months)/2.

MD= +15.78 months, MD + 2 SD = +40.93 months, MD – 2SD = -9.37 months.

**Table-I:** Differences and relationship between chronological age (CA) and bone age by Greulich & Pyle Atlas (GP) in different genders.* indicate significant at p<0.05

*Gender*		*N*	*Paired Samples T Test*					*Pearson’s Correlation*	
			*Mean difference* *(months)*	*Std. Deviation of difference* *(months)*	*95% CI of the Difference* *(months)*		*p value*	*r*	*p value*
					*Lower*	*Upper*			
Female	CA - GP	81	6.6543	13.4733	3.6751	9.6335	0.000*	0.788	0.000*
Male	CA - GP	139	15.7842	12.8304	13.6323	17.9360	0.000*	0.816	0.000*

**Table-II:** Differences between chronological age (CA) and bone age by Greulich & Pyle Atlas (GP) in different age quartiles.* indicate significant at p<0.05

*Gender*	*Ages(months)*		*N*	*Paired Differences*				
				*Mean Difference* *(months)*	*Std. Deviation of difference* *(months)*	*95% CI of the Difference*		*p value*
						*Lower* *(months)*	*Upper* *(months)*	
Female	54-65	CA – GP	16	4.4375	15.1260	-3.6226	12.4976	0.259
	66-77	CA – GP	16	8.9375	13.4088	1.7925	16.0825	0.018*
	78-89	CA – GP	21	6.0000	14.5465	-.6215	12.6215	0.073
	90-101	CA – GP	15	8.2667	12.7418	1.2105	15.3228	0.025*
	102-113	CA – GP	13	5.7692	11.7909	-1.3559	12.8944	0.103
Male	54-65	CA – GP	37	13.1892	10.7104	9.6182	16.7602	0.000*
	66-77	CA – GP	27	13.8889	13.6222	8.5001	19.2776	0.000*
	78-89	CA – GP	23	18.8696	13.4039	13.0733	24.6658	0.000*
	90-101	CA – GP	26	16.5769	11.9839	11.7365	21.4173	0.000*
	102-113	CA – GP	26	17.9231	14.8591	11.9213	23.9248	0.000*

A mean difference of less than 6 months is noted between the chronological age and bone age of females aged 54-65 months, 78-89 months and 102-113 months. However, mean differences of more than 8 months are noted in females aged 66-77months and 90-101 months. (Table-II).

A mean difference of more than 13 months is observed between chronological age and bone age in all male age groups (minimum mean difference 13.9 months and maximum mean difference 18.9 months). (Table-II).

## DISCUSSION

Our study indicates that there is a great variation in the chronological age and bone age calculated by the Greulich & Pyle Atlas. The degree of disparity between the two ages is less in females, with mean differences ranging from 4.4 to 8.9 months (Table-II) whereas it is markedly higher in males ranging from 13.2 to 18.9 months (Table-II).

Various international studies have reported different results regarding the applicability of the Greulich & Pyle Atlas for estimation of chronological age. In Australia, bone ages of males are on average advanced by 0.4 years, whereas bone ages of females on average are skeletally delayed by 0.3 years when using this atlas.^14^Another study found significant difference in chronological and bone ages in Israeli boys.^9^However, statistically significant difference between the means and standard deviations of up to one year have been reported between chronological age and bone age of Turkish chidren.^15^

There is a paucity of literature from Pakistan regarding bone age assessment in children. However, our results are consistent with Shaikh et al^13^ who performed a similar study on children aged 8-18 years at Chandka Medical College, Larkana, and reported a mean differences of ages in females and males as 0.5 and one years respectively. Zafar et al^12^ who compared bone and chronological ages at the Agha Khan University Hospital, Karachi also found that the mean differences between the ages was less in females as compared to males in children of middle and late childhood.

The scatterplot graphs show that difference between chronological age and bone age by Greulich and Pyle method in 95% of females range from -19.75 months to +33.05 months (Fig.1) and in 95% of males range from -9.37 months to +40.93 months(Fig.2). This extreme discrepancy makes the Greulich & Pyle Atlas invalid for forensic application of bone age in the estimation of chronological age in Pakistani children.

However there is a high correlation between the two ages in both genders (Table-I), which makes serial measurements of bone age useful in diagnosing and treating endocrine disorders of growth and stature.

## CONCLUSION

Bone age calculated by Greulich & Pyle Atlas significantly underestimates chronological age in Pakistani children between the ages of 54-113 months. The mean difference between chronological age and bone age calculated by Greulich & Pyle Atlas is greater in boys compared to girls. This significant difference between the two ages limits use of Greulich & Pyle Atlas in estimating chronological age for medico legal purposes. However, a high correlation between chronological and bone age by Greulich & Pyle Atlas makes it suitable for followup use in growth disorder patients.

## Notes

***Source of funding:*** Ziauddin University.
